# Pre‐operative and prehabilitation services in UK cardiac surgery centres

**DOI:** 10.1111/anae.15918

**Published:** 2022-11-17

**Authors:** B. Gibbison, G. J. Murphy, E. Akowuah, M. Loubani, M. Pufulete

**Affiliations:** ^1^ Bristol Medical School University of Bristol UK; ^2^ University of Leicester UK; ^3^ South Tees NHS Foundation Trust Newcastle UK; ^4^ Hull York Medical School Hull UK

Prehabilitation is the practice of enhancing a patient's functional capacity before surgery, with the aim of improving postoperative outcomes [1]. Research into prehabilitation was identified as one of the top 10 priorities for cardiac surgery [2]. Cardiac surgery has two distinct treatment pathways; approximately half of the 32,000 adult cardiac surgery episodes in the UK per year are planned (mean waiting time of 104 days) and half are urgent inpatients (mean waiting time of 11 days), plus a small number of emergency cases [3]. In order to maximise the potential for improved patient outcomes, prehabilitation in both pathways must be addressed. This survey was designed to understand how pre‐operative cardiac surgical pathways are structured across the UK, and to what extent prehabilitation is implemented.

An online survey was developed and piloted by a multidisciplinary research team (online Supporting Information, Appendix S1). All 35 centres performing cardiac surgery in the UK were contacted and sent the online link (SurveyMonkey). Sites were defined by geography rather than NHS Trust and the most appropriate person (anaesthetist, surgeon or service manager) was identified and asked to complete the survey.

Thirty‐four of the 35 cardiac surgery centres (97%) responded to the survey. The results are shown in Table 1. In 21/34 centres (62%) there is a dedicated senior nurse in charge of pre‐operative assessment (POA) and only 16/34 (47%) have a nominated medical lead. Most centres (23/29, 93%) assess < 50 patients per week and most patients are assessed face‐to‐face. For elective patients, about half the centres offer POA between 1 and 4 weeks before surgery. One centre stated that there is currently no POA infrastructure.

**Table 1:** Pre‐operative assessment (POA) service and prehabilitation components in urgent and elective cardiac surgery pathways from 34 cardiac surgery centres. Values are number (proportion).

About the POA service	Number of centres
POA has nominated clinician lead	
Yes	16 (47%)
POA has a dedicated senior nurse in charge	
Yes	21 (62%)
Number of nursing staff full‐time equivalents in POA	
> 5	5 (15%)
3–5	11 (32%)
1–2	10 (29%)
Do not know	8 (24%)
Number of patients assessed in POA per week	
> 100	1 (3%)
50–99	1 (3%)
< 50	26 (77%)
Do not know	6 (18%)
Proportion of patients attending POA living within 10 miles of the hospital	
> 75%	4 (12%)
50–74%	2 (3%)
25–49%	6 (21%)
< 25%	5 (15%)
Do not know	17 (49%)
Elective patients undergo POA	n = 33
On day of consultation with the cardiac surgeon	2 (6%)
> 4 weeks before surgery	7 (21%)
1–4 weeks before surgery	17 (52%)
≤ 1 week before surgery	0
On admission for surgery	2 (6%)
Other^a^	5 (15%)
Proportion of elective patients assessed face to face	n = 33
100%	17 (52%)
75%	3 (9%)
50%	2 (6%)
25%	2 (6%)
Do not know	9 (27%)
Urgent patients undergo POA	n = 33
In the referring hospital / unit	3 (9%)
On admission to the cardiac surgery unit	22 (67%)
Both the above	1 (3%)
Other^b^	7 (21%)
Patient assessment according to a proforma	n = 33
Yes	21 (64%)

**Data collection for service audit, quality assurance or improvement framework**	**n = 33**
Clinical outcome data (e.g. mortality, complications, length of stay, readmission to hospital, etc.)	23 (70%)
Patient‐reported outcome data (e.g. patient satisfaction, quality of life, etc.)	8 (24%)
POA service not currently audited	9 (27%)
Other	3 (9%)
**Prehabilitation**	
Offered to patients undergoing cardiac surgery	n = 33
Yes	4 (12%) (elective only)
Offered to other surgical specialties in the Trust	n = 29
Yes	19 (17%)
Do not know	5 (17%)
Planned for cardiac surgery	n = 29
Yes	16 (55%)
No	9 (31%)
Do not know	4 (14%)

^a^ A mixture of the above (n = 1); there is currently no infrastructure (n = 1); no dedicated timeframe (exact timing varies) (n = 1).

^b^ No POA (n = 3); day before surgery (n = 2).

For patients who require urgent surgery, 22 (67%) centres offer POA on admission to the cardiac surgery unit, although three (10%) have no POA for the urgent pathway and any POA is performed in the referring hospital.

Only four cardiac surgery centres (13%) reported offering any prehabilitation to their patients (and only on the elective pathway) despite prehabilitation being offered within other surgical specialties by 19/29 (66%) of NHS Trusts represented in the survey. One centre offers psychological and one centre offers exercise prehabilitation only. One centre offers multimodal prehabilitation, including an exercise prescription, respiratory exercises, a dietary intervention (oral nutritional supplements), psychological support and patient education. One centre offers a similar package, but without the psychological component. In all four centres, prehabilitation is initiated at POA or the outpatient appointment following surgical consultation. The service is funded in only one centre. None of the four centres audit their prehabilitation programme.

This survey shows that less than half the cardiac surgical centres in the UK offer robust pre‐operative assessment to their elective patients with a nominated medical lead and senior pre‐operative nurse. This is at odds with the Royal College of Anaesthetists' General Provision of Anaesthetic Services guidelines regarding pre‐operative assessment [4]. There is little offer of prehabilitation to elective cardiac surgical patients and no offer within the urgent pathway, although half the UK centres have a plan to set one up. Cardiac surgery case‐mix has changed over the last two decades with an increasing number of older adults (including those aged ≥ 70 y) undergoing valve surgery [3]. These patients have increased comorbidities and frailty, which often need managing in the peri‐operative period.

Our survey demonstrates that although there is wide interest in prehabilitation for cardiac surgery, there is little application of this for cardiac surgery patients (elective or urgent). This is despite prehabilitation being offered to other surgical cohorts in the same hospital. There are guidelines for prehabilitation in major cancer [5] and joint replacement surgery [6]. Cardiac surgery, although major, is considered different to non‐cardiac surgery with regards to prehabilitation because of the high proportion of patients undergoing urgent surgery in which there is considered to be insufficient time for prehabilitation to have any effect and concerns over exercising patients with stenotic lesions (e.g. aortic stenosis, left main stem coronary artery disease). Cardiac surgery is a tertiary service and patients must sometimes travel considerable distances, making in‐person and repeat visit education programmes challenging [7].

A limitation of this survey is that prehabilitation is not universally defined and, as such, some centres may be implementing components of prehabilitation (e.g. nutritional advice, psychological support) without labelling them ‘prehabilitation’. In addition, as > 50% of centres have no medical leadership in POA, the individual answering the survey may not be fully apprised of all the pre‐operative services.

We aim to develop a prehabilitation intervention that can be implemented within UK cardiac surgery pathways. Despite the implementation of prehabilitation in other major surgical specialities, there is little evidence that it improves clinical outcomes [8]. The late adoption of pre‐operative assessment and prehabilitation by cardiac surgery offers the opportunity of ensuring that any interventions are fit‐for‐purpose and cost‐effective for the healthcare system.

## Supporting information


**Appendix S1.** Online survey.
