# Supplementary material for: Pre‐operative and prehabilitation services in UK cardiac surgery centres
Source: Anaesthesia. 2022 Nov 17;78(3):388–91. doi: 10.1111/anae.15918 (PMC10099756; doi:10.1111/anae.15918)
Supplement: Supplementary file 1 — Appendix S1. Online survey. [file ANAE-78-388-s001.pdf]

### What this survey is about

**This survey is part of a research study entitled *Prehabilitation before cardiac surgery in the UK (CARDIAC PREHAB-UK)*. We want to find out the current preoperative assessment (POA) practice for cardiac surgery patients in your centre, whether you offer a prehabilitation programme to your patients and what this entails.**

### Completing the survey

**The survey will take about 10 minutes to complete. Your responses will be kept safe and secure. All data will be stored in password protected electronic format at the University of Bristol. The results of this study will be used for research purposes and only amalgamated results will be presented. You will not be identified in any output from this research.**

### Additional information

**The study is funded by the National Institute for Health and Care Research (NIHR) Programme Development Grants programme (project reference NIHR127850). The views expressed are those of the author(s) and not necessarily those of the NIHR or the Department of Health and Social Care.**

**If you have any questions about the research study, please contact Dr Ben Gibbison at ben.gibbison@bristol.ac.uk.**

Name of your Hospital / Trust / Board

Your name and email address

Your role

Is there a nominated clinician lead for POA?

- ◯ Yes
- ◯ No

Is there a dedicated senior nurse in charge of POA?

- ◯ Yes
- ◯ No

Approximately how many full time equivalent (FTE) nursing staff do you currently have working in POA?

- ◯ >5
- ◯ 3-5
- ◯ 1-2
- ◯ Don't know

Approximately how many patients are assessed in the POA Clinic each week?

- ◯ >100
- ◯ 50-99
- ◯ <50
- ◯ Don't know

Approximately what proportion of patients attending POA live within 10 miles of the hospital?

- ◯ >75%
- ◯ 50-74%
- ◯ 25-49%
- ◯ <25%
- ◯ Don't know

Is there anything else you would like to say about POA for cardiac surgery patients in your hospital? Please write it here

## SECTION B: PATIENT ASSESSMENT

In the **ELECTIVE** cardiac surgery pathway, at which point do patients undergo POA?

- ◯ Immediately after consultation with the cardiac surgeon (same day)
- ◯ >4 weeks before surgery
- ◯ 1 - 4 weeks before surgery
- ◯ ≤1 week before surgery
- ◯ On admission for surgery
- ◯ Other (please specify)

In the **URGENT** cardiac surgery pathway, at which point do patients undergo POA?

- ◯ In the referring hospital / unit
- ◯ On admission to the cardiac surgery unit
- ◯ Other (please specify)

In the **ELECTIVE** POA Clinic, approximately what proportion of patients are assessed face to face (rather than by telephone or virtually)?

- ◯ 100%
- ◯ 75%
- ◯ 50%
- ◯ 25%
- ◯ Don't know

Regarding **ELECTIVE AND URGENT** POA: Is the patient assessment “protocol driven” (i.e., according to a proforma)?

- ◯ Yes
- ◯ No

Do your routinely screen pre-operatively for? *Please tick all that apply*

- ☐ Anaemia
- ☐ Poor diabetic control (HbA1c > 69 mmol/mol)
- ☐ Undiagnosed sleep apnoea
- ☐ None of the above
- ☐ Other (please specify)

Do you undertake the following assessments routinely? *Please tick all that apply*

- [ ] Physical fitness (e.g., CPET testing / incremental shuttle walk test)
- [ ] Nutrition (e.g., weight loss, poor food intake, body mass index, body composition)
- [ ] Psychological (e.g., anxiety, depression)
- [ ] Frailty
- [ ] Smoking/ alcohol intake
- [ ] Cognitive impairment
- [ ] None of the above
- [ ] Other (please specify)

Do you have a standard pathway for the optimisation of patients identified to have? *Please tick all that apply*

- [ ] Poorly controlled diabetes (Hba1c > 69)
- [ ] Anaemia
- [ ] Poorly managed respiratory disease
- [ ] Frailty
- [ ] None of the above
- [ ] Other (please specify)

Do you refer patients to other services for optimisation before surgery? *Please tick all that apply*

- [ ] Smoking cessation
- [ ] Alcohol liaison
- [ ] Dietetics
- [ ] Physiotherapy
- [ ] Clinical psychology
- [ ] Occupational therapy
- [ ] Rehabilitation/therapy support
- [ ] None of the above
- [ ] Other (please specify)

Do you collect any of the following as part of a service audit, quality assurance or improvement framework? *Please tick all that apply*

- ☐ Clinical outcome data (e.g., mortality, complications, length of hospital / intensive care stay, readmission to hospital, etc.)
- ☐ Patient-reported outcome data (e.g., patient satisfaction, quality of life, etc.)
- ☐ The service is not currently audited
- ☐ Other (please specify)

## SECTION C: PREHABILITATION

Do you have a prehabilitation programme for cardiac surgery patients?

- ○ Yes
- ○ No

## SECTION C: PREHABILITATION

The prehabilitation programme is being offered to patients undergoing

*Please tick all that apply*

- ☐ Any cardiac surgery
- ☐ CABG only
- ☐ Valve only
- ☐ Elective patients only
- ☐ Elective and urgent patients
- ☐ None of the above

What does your prehabilitation programme include?

*Please tick all that apply*

- ☐ Exercise prescription
- ☐ Respiratory exercise (e.g., inspiratory muscle training, incentive spirometry)
- ☐ Oral nutritional supplements
- ☐ Dietary intervention to reverse malnutrition (e.g., obesity, sarcopenia, frailty)
- ☐ Psychological support
- ☐ Education (to improve patient knowledge, self-efficacy and resilience)
- ☐ Cognitive impairment intervention
- ☐ None of the above
- ☐ Other (please specify)

How is your prehabilitation programme delivered?

*Please tick all that apply*

- ☐ In hospital
- ☐ In the community
- ☐ Phone or video sessions
- ☐ Online live group sessions
- ☐ Resources provided for self-delivery
- ☐ Interactive App
- ☐ None of the above

Please provide further detail of your prehabilitation programme here

Has the delivery of your prehabilitation programme changed due to the COVID-19 pandemic?

- ◯ Yes
- ◯ No

If yes, please state how

[ ]

Which of the following clinical specialties are involved in delivering your prehabilitation programme?

*Please tick all that apply*

- ☐ Anaesthetists
- ☐ Surgeons
- ☐ Clinical nurse specialists
- ☐ Dietitians
- ☐ Physiotherapists
- ☐ Exercise instructors
- ☐ Occupational therapists
- ☐ Rehabilitation/therapy support staff
- ☐ Exercise physiologists
- ☐ Clinical psychologists
- ☐ Other (please specify)

[ ]

At which point in the treatment pathway are patients referred for prehabilitation?

*Please tick all that apply*

- ☐ Outpatient appointment following surgical consultation
- ☐ Pre-operative assessment
- ☐ Other (please specify)

[ ]

Do you use any of the following to assess patient adherence / engagement with the prehabilitation programme?

*Please tick all that apply*

- [ ] Patient diaries
- [ ] Regular communication with patients (email, telephone, via app or video consultation)
- [ ] Patient attends the hospital regularly during the programme
- [ ] We do not currently collect patient adherence data
- [ ] Other (please specify)

Who funds your organisation's prehabilitation service?

*Please tick all that apply*

- [ ] Commissioned service
- [ ] Charity
- [ ] Part of a research study
- [ ] The service is not funded as a prehabilitation service
- [ ] Don't know
- [ ] Other (please specify)

Is there a prehabilitation programme for other surgical specialties in your trust (e.g., orthopaedics or cancer)

- Yes
- No
- Don't know

Are you planning to set up a prehabilitation service for cardiac surgery patients in your trust?

- Yes
- No
- Don't know

Comments

Any other comments? If you have anything else you would like to say about prehabilitation in cardiac surgery patients, please write it here

**Thank you for completing this survey.**
